# Screening of Anti-Hair Loss Plant Raw Materials Based on Reverse Network Pharmacology and Experimental Validation

**DOI:** 10.3390/cimb47010068

**Published:** 2025-01-20

**Authors:** Jiajia Xu, Congfen He, Rui Tian

**Affiliations:** Beijing Key Laboratory of Plant Resources Research and Development, School of Light Industry Science and Engineering, Beijing Technology and Business University, Beijing 100048, China; xujj0909@163.com (J.X.); tian4641750@163.com (R.T.)

**Keywords:** hair loss, reverse network pharmacology, Wnt/β-catenin signaling pathway, TGFβ/BMP signaling pathway, lipids, licorice, salvia miltiorrhiza, mulberry leaf, ephedra, curcumae radix

## Abstract

Hair loss is one of the skin conditions that can affect people’s mental health. Plant raw material extracts are of great interest due to their safety. In this study, we utilize reverse network pharmacology to screen for key targets of the Wnt/β-catenin signaling pathway and the TGFβ/BMP signaling pathway, as well as key differential lipids, for plant raw materials selection. The aim is to identify plant raw materials that may have anti-hair loss properties and to validate these findings through cell experiments. Licorice, salvia miltiorrhiza, mulberry leaf, ephedra and curcumae radix were found that may possess anti-hair loss effects. Licorice water extract (LWE), salvia miltiorrhiza water extract (SMWE), mulberry leaf water extract (MLWE), ephedra water extract (EWE) and curcumae radix water extract (CRWE) did not exhibit cytotoxicity on human dermal papilla cells (HDPCs). Through ALP staining, it was found that the expression of ALP in HDPCs treated with LWE, SMWE, MLWE, EWE and CRWE was enhanced. In addition, LWE, SMWE, MLWE, EWE and CRWE have reduced the expression of hair growth inhibitory factor TGF-β1 and inflammatory factor IL-6. Additionally, various water extracts can enhance the secretion of VEGF, with high concentrations of SMWE, EWE and CRWE exhibiting better efficacy. Furthermore, β-catenin, a key factor of the Wnt/β-catenin signaling pathway, was enhanced by LWE, SMWE, MLWE, EWE and CRWE treatment in cultured HDPCs. In conclusion, all five plant raw materials showed some anti-hair loss potential, providing theoretical support for their application in anti-hair loss products.

## 1. Introduction

Hair is a significant aspect of a person’s overall appearance, and healthy hair symbolizes their unique personality. Hair loss, characterized by thinning hair or a receding hairline, can negatively impact an individual’s self-confidence and may lead to various psychological issues, including anxiety and depression [1,2].

The Wnt/β-catenin signaling pathway is one of the most important signaling pathways for hair follicle development, and various Wnt ligands have promoted the hair growth cycle and hair regrowth by activating β-catenin [3]. Zhou et al. found that the overexpression of Wnt10b leads to the activation of the Wnt/β-catenin signaling pathway, which in turn enhances the proliferation and migration of outer root sheath cells (ORCs) [4]. Furthermore, the upregulation of Wnt3a and Wnt5a accumulated β-catenin and activated the Wnt/β-catenin signaling pathway, which led to a marked increase in the number and size of hair follicles in the mice [5]. The TGF-β family, which includes TGF-β and bone morphogenetic proteins (BMP), serves as a critical regulator of cell proliferation and differentiation. TGF-β facilitates the transition of hair follicles to the regressive phase by inhibiting cell proliferation and inducing apoptosis, whereas BMP is essential in orchestrating the initiation of the anagen phase in hair follicle cycling [6,7]. Regulation of the TGF-β/BMP signaling pathway significantly augments the number of hair follicles in murine models [8]. The downregulation of TGF-β1 stimulates keratinocyte (KC) proliferation and inhibits apoptosis, thereby ameliorating alopecia [9]. Furthermore, BMP6 exerts an inhibitory effect on the activation of hair follicle stem cells and the transition from telogen to anagen in hair follicle cycling in mice [10]. Lipids play structural roles and regulatory roles in the hair follicle. Comprising 0.7% to 1.3% of the total hair composition, they are crucial for maintaining hair integrity [11,12]. Additionally, lipids play a significant role in safeguarding the hair against environmental and chemical damage, and they also act as a barrier to prevent moisture loss [13]. Our group previously conducted a comparative lipidomics analysis of hair root lipids between alopecia patients and healthy controls, identifying 45 differentially expressed lipids. Among these, 10-oxo-nonadecanoic acid, myriocin, Cer(d18:0/22:0), Cer(d18:0/24:0), Cer(d18:0/18:0) and Cer(d18:0/16:0) demonstrated AUC greater than 0.7, suggesting their potential as diagnostic biomarkers or therapeutic targets for alopecia [14].

Currently, the primary pharmacological interventions for hair loss are minoxidil (MNX) and finasteride (FINA). However, these treatments exhibit several drawbacks, including dependency issues, such as hair regrowth following discontinuation of the medication. Additionally, long-term use may lead to potential scalp irritation or allergic reactions, and there is a possibility that these treatments could disrupt the normal hair growth cycle [15,16]. In recent years, with people’s pursuit of natural, safe and healthy products, the use of plant raw material extracts in the treatment of hair loss has become increasingly widespread. The advantages of plant extracts as anti-hair loss raw materials are mainly reflected in their high safety, mild effect and long-lasting effects [17,18].

Network pharmacology, which integrates systems biology and bioinformatics, analyzes the molecular associations between drugs and therapeutic targets to reveal the systemic pharmacological mechanisms of drugs [19]. This approach is widely used in the investigation of the mechanisms underlying the treatment of diseases with herbal medicine, with reverse network pharmacology being one of its significant branches [20,21]. Herbal medicines have the characteristics of multi-target and multi-pathway regulation. Reverse network pharmacology adopts a “targets → compounds → herbal medicines” approach, which enables large-scale screening of compounds from multiple disease targets to discover plant materials with therapeutic potential for treating the disease [21,22].

This study focuses on the targets of key anti-hair loss pathways, namely the Wnt/β-catenin and TGFβ/BMP signaling pathways, as well as key differential lipids, to search for plant raw materials. Utilizing reverse network pharmacology, we systematically screen and validate effective components for hair loss prevention from multiple perspectives, with the aim of identifying plant raw materials most likely to be effective in preventing hair loss.

## 2. Materials and Methods

### 2.1. Reverse Collection of Active Ingredients from Key Pathway Targets

The names of the target proteins were entered individually into the HERB database (http://herb.ac.cn/ (accessed on 16 November 2023)) to search for active ingredients associated with these proteins. The active ingredients were then filtered based on the conditions of having a PubChem ID, a molecular weight of 500 or less and complying with the Lipinski’s Rule of Five.

### 2.2. Reverse Collection of Targets of Action and Active Ingredients from Key Differential Lipids

For the six differential lipids related to hair loss that were previously identified by the research group, the SMILES format of these lipids was obtained from the LIPID MAPS database (https://lipidmaps.org/ (accessed on 11 December 2023)). The SMILES format of the lipids was then input into the Swiss Target Prediction website (http://swisstargetprediction.ch/ (accessed on 11 December 2023)). After retrieving the relevant targets of the lipids, these targets were filtered based on the criterion of probability* > 0. Using ‘Alopecia’ as the keyword, the GeneCards database (https://www.genecards.org/ (accessed on 12 December 2023)) was searched to identify relevant targets. The intersection of the two targets was plotted as a Venn diagram to obtain the intersecting targets. Finally, active ingredients were collected based on these targets, following the same method as in Section 2.1.

### 2.3. Construction of Key Pathway Targets–Active Ingredients–Plant Raw Materials Network

Plant raw materials that contain active ingredients that met the screening criteria were collected from the HERB database. Subsequently, the targets–active ingredient–plant raw material network diagram was constructed using Cytoscape (3.9.1).

### 2.4. Construction of Key Differential Lipids–Targets–Active Ingredients–Plant Raw Materials Network

Plant raw materials that contain active ingredients that met the screening criteria were collected from the HERB database. Subsequently, the lipids–targets–active ingredients–plant raw material network diagram was constructed using Cytoscape.

### 2.5. Sample Preparation

Licorice tablets, Salvia miltiorrhiza tablets, mulberry leaf tablets and curcumae radix tablets were purchased from Jiuzhou Shanghekuan Pharmacy (Wuhan, China) and ephedra tablets were purchased from Renxintang pharmacy (Shenzhen, China). Licorice tablets, Salvia miltiorrhiza tablets, mulberry leaf tablets, ephedra tablets and curcumae radix tablets were crushed and sieved through an 80-mesh sieve. The extraction was performed using ultrasonic extraction at 60 °C for 1 h, following a material-to-liquid ratio of 1:10 (g:mL), repeated twice. The supernatant was collected after centrifugation and concentration and then lyophilized to obtain the aqueous extract powder. Then, take 10 mg of the water extract powder and dissolve it in 10 mL of deionized water, making the concentration of 1 mg/mL for the water extract.

### 2.6. Cell Culture

Human dermal papilla cells (HDPCs) were purchased from NEWGAINBIO (Wuxi, China). HDPCs were cultured in high-sugar DMEM (Cellmax, Beijing, China) supplemented with 10% FBS (NEWGAINBIO, Wuxi, China), 100 U·mL^−1^ penicillin G and 100 µg·mL^−1^ streptomycin 1% double antibody (Gibico, Carlsbad, CA, USA). Cells were maintained in a humidified incubator at 37 °C with 5% CO_2_. When the cells grew adherently to a confluence greater than 80%, they were digested with trypsin and collected by centrifugation at 1000 rpm for 4 min, then passaged at a ratio of 1:2. Cells in the logarithmic growth phase were used for subsequent experiments.

### 2.7. Cell Viability Assay

CCK-8 assay (Beyotime, Shanghai, China) was used to detect the effects of Licquorice water extract (LWE), salvia miltiorrhiza water extract (SMWE), mul-berry leaf water extract (MLWE), ephedra water extract (EWE) and curcumae radix water extract (CRWE) on the viability of HDPCs. Logarithmically growing HDPCs were adjusted to a density of 1 × 10^4^ cells per well and inoculated into 96-well plates, with 100 μL per well. The plates were then placed in a cell culture incubator at 37 °C with 5% CO_2_ for 24 h. Control and experimental groups were set up, respectively, and different concentrations of water extract were added to the experimental group. Each group had 3 replicates and was incubated in the cell culture incubator for 24 h. The absorbance at 450 nm was read using a microplate reader.

### 2.8. Alkaline Phosphatase (ALP) Staining Assay

The ALP staining kit was acquired from Beyotime (Shanghai, China). Logarithmically growing HDPCs were adjusted to a density of 3 × 10^4^ cells per well and inoculated into 24-well plates, with 500 μL per well. The supernatant was discarded after 24 h of incubation in a cell culture incubator. Add fresh culture medium with different concentrations of water extract solutions according to the experimental groups. After incubating for 72 h, staining occurred according to the kit instructions and capture images using a microscope.

### 2.9. ELISA Assay

Logarithmically growing HDPCs were adjusted to a density of 7 × 10^4^ cells per well and inoculated into 12-well plates, with 1 mL per well. The supernatant was discarded after 24 h of incubation in a cell culture incubator. According to the experimental groups, 1 mL of fresh culture medium containing different concentrations of the water extract solution was added. To the blank control 1 mL of fresh medium was added, while to the positive control group 1 mL of fresh medium containing 5 μM and 10 μM MNX was added. After continuing the culture for 48 h, the supernatant, with 3 replicates set for each group was collected, and an ELISA kit (Cusabio, Wuhan, China) was utilized to measure the VEGF content.

### 2.10. Real-Time Quantitative PCR (RT-qPCR)

Gene transcription was quantified by RT-qPCR. Collect cells from each group and extract total RNA according to the kit’s instructions (Omega Bio-Tek, Norcross, GA, USA). Determine the concentration and quality of the RNA and subsequently perform reverse transcription to cDNA following the kit instructions (Accurate Biology, Changsha, China). RT-qPCR experiments were conducted using a three-step protocol, with GAPDH serving as the internal reference. Relative gene expression was calculated by 2^−ΔΔCT^ method. The sequences of the primers utilized are presented in Table 1.

### 2.11. Western Blot Assay

Collect cells from each group and lyse them on ice using RIPA buffer (Beyotime, Shanghai, China) containing PMSF (Beyotime, Shanghai, China). Centrifuge the lysate to obtain the supernatant, then take an appropriate amount of the sample to determine protein concentration using the BCA assay (Beyotime, Shanghai, China). Add the remaining protein to 5× loading buffer (Beyotime, Shanghai, China). After heating the mixture in a metal bath at 95 °C for 5 min, store it at −80 °C. Following separation of the proteins on a 10% SDS-PAGE gel (Epizyme, Shanghai, China), transfer them to a PVDF membrane (Epizyme, Shanghai, China). Block the membrane with 5% skimmed milk, then incubate it overnight at 4 °C with a specific primary antibody (β-catenin) (Proteintech, IL, USA). Wash the membrane three times with TBST (Biosharp, Anhui, China), allowing 8 min for each wash, and then incubate it with a diluted secondary antibody (Immunoway, San Jose, CA, USA) at room temperature for 90 min. Use ECL reagent (Mi Mouse, Xi’an, China) for luminescent imaging. Employ GAPDH as an internal control and analyze the intensity of the bands using ImageJ software version 1.8.0.

### 2.12. Statistical Analysis

Statistical analysis was conducted using GraphPad Prism (9.5, GraphPad Software, San Diego, CA, USA), and the data are presented as “mean ± standard deviation”. Comparisons between groups were analyzed using Student’s *t*-test and visualized with GraphPad Prism A *p*-value of <0.05 was considered statistically significant, <0.01 was deemed highly statistically significant, and <0.001 was regarded as extremely statistically significant.

## 3. Results

### 3.1. Reverse Collection of Active Ingredients from Key Pathway Targets

Based on 17 targets from the Wnt/β-catenin signaling pathway and the TGFβ/BMP signaling pathway—specifically, β-catenin, AXIN2, LEF-1, GSK-3β, Wnt3a, Wnt4, Wnt5a, Wnt7b, Wnt10a, Wnt10b, DKK1, BMP2, BMP4, BMP6, TGF-β1, TGF-β2 and TGF-β3—a total of 941 active components were screened. The information regarding the top eight active components, ranked by degree, is shown in Table 2.

### 3.2. Reverse Collection of Targets of Action and Active Ingredients from Key Differential Lipids

**Figure 1:**
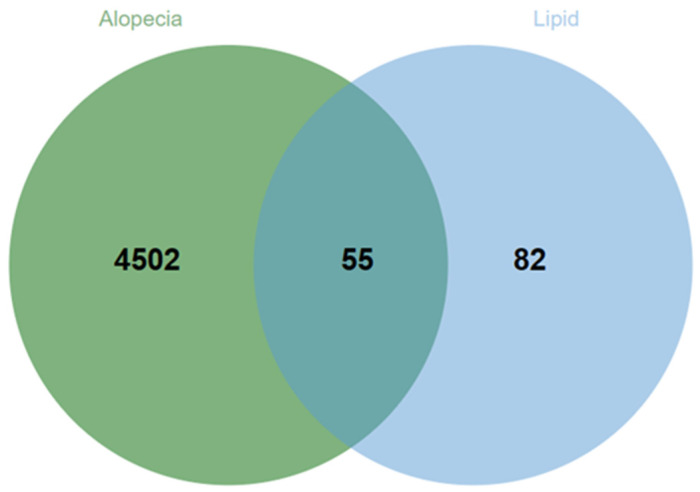
Venn diagram of intersecting targets of key differential lipids and alopecia.

A total of 137 targets were screened based on key differential lipids. By intersecting these with the 4557 hair loss-related targets obtained from the GeneCards database, we identified 55 targets associated with hair loss, as shown in Figure 1. Among these, 53 targets can be found in the HERB database, as listed in Table S1. A reverse screening identified 3562 active components, with the top eight active components ranked by degree as shown in Table 3.

### 3.3. Construction of Targets–Active Ingredients–Plant Raw Materials Network

#### 3.3.1. Construction of Key Pathway Targets–Active Ingredients–Plant Materials Network

**Figure 2:**
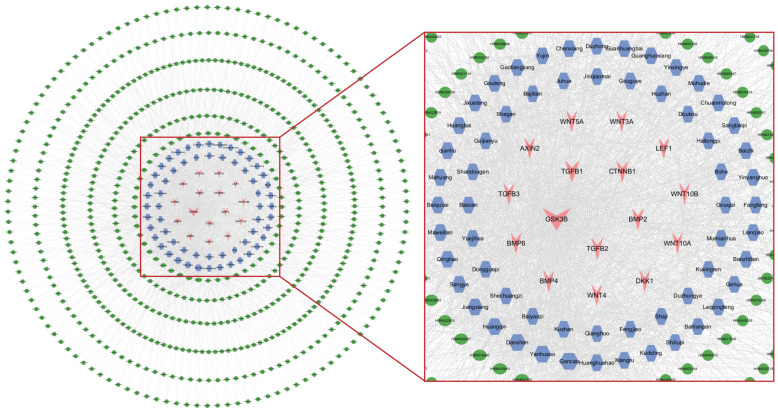
Key targets–active ingredients–plant raw materials network (plant raw materials degree ≥ 60). The green nodes represent active ingredients, the blue nodes represent plant raw materials, and the red nodes represent targets. The edges represent the interactions between them, and node sizes are proportional to their degree.

A total of 941 active ingredients information was collected in HERB, along with 1024 plant materials. The top 60 plant raw materials, ranked by degree value, were selected to construct a key targets–active ingredients–plant raw materials network, as shown in Figure 2. The plant raw materials ranked in the top 17 by degree value are listed in Table 4.

#### 3.3.2. Construction of Targets–Active Ingredients–Plant Raw Materials Network Based on Key Differential Lipids

**Figure 3:**
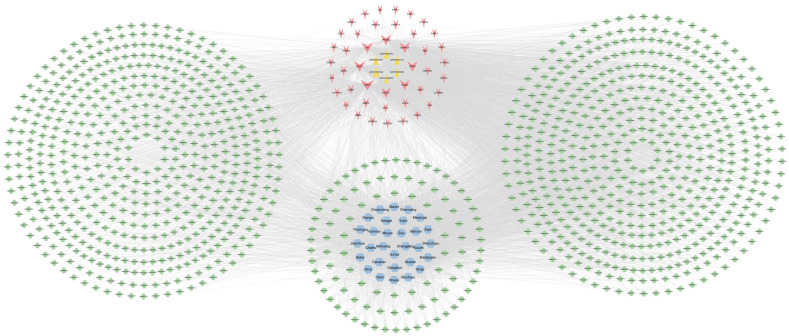
Lipids–key targets–active ingredients–plant raw materials network (plant raw materials degree ≥ 30). The green nodes represent active ingredients, the blue nodes represent plant raw materials, red nodes represent targets, and the yellow nodes represent lipids. The edges represent the interactions between them, and node size are proportional to their degree.

A total of 3562 active ingredient information was collected in HERB, along with 3485 plant raw materials. The top 30 plant raw materials, ranked by degree value, were selected to construct a key targets–active ingredients–plant raw material network, as shown in Figure 3. The plant raw materials ranked in the top 17 by degree value are listed in Table 5.

### 3.4. Identification of Key Plant Materials

**Figure 4:**
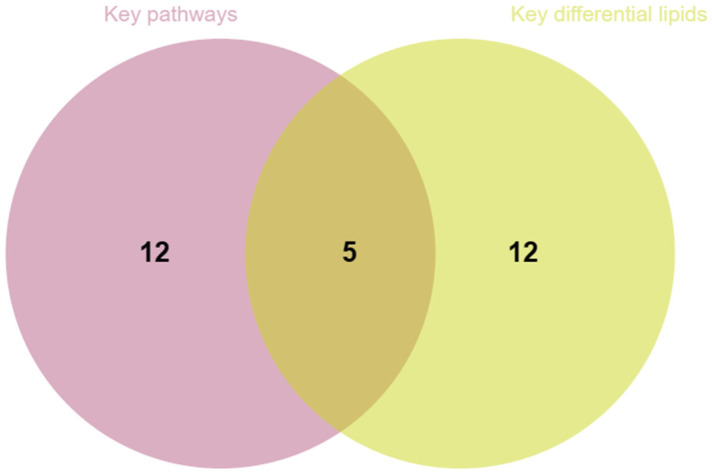
Venn diagram of intersecting plant raw materials of key differential lipids and key pathways.

Based on the key pathways and the key differential lipids, a comprehensive screening of plant raw materials was conducted. As shown in Figure 4, five plant materials with the potential for preventing hair loss were identified: licorice, Salvia miltiorrhiza, mulberry leaf, ephedra and curcumae radix.

### 3.5. Effect of LWE, SMWE, MLWE, EWE and CRWE on the Cell Viability of HDPCs

**Figure 5:**
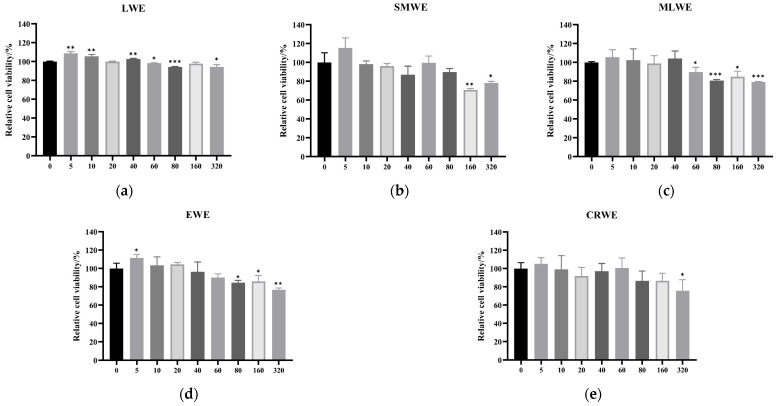
The effect of (**a**) LWE, (**b**) SMWE, (**c**) MLWE, (**d**) EWE and (**e**) CRWE at 0, 5, 10, 20, 40, 60, 80, 160 or 320 μg/mL on the cell viability of HDPCs was measured by CCK-8 assay. Results are presented as mean ± standard deviation of the mean. * *p* < 0.05, ** *p* < 0.01 and *** *p* < 0.001 vs. control group. LWE, licquorice water extract; SMWE, salvia miltiorrhiza water extract; MLWE, mulberry leaf water extract; EWE, ephedra water extract; CRWE, curcumae radix water extract.

To investigate the effect of water extracts with different concentrations on the viability of HDPCs, the CCK-8 method was used to assess the impact of LWE, SMWE, MLWE, EWE and CRWE on the viability of HDPCs. As shown in Figure 5, the cell viability of different water extracts, ranging from 5 μg/mL to 320 μg/mL, exceeds 70%. Overall, none of the individual water extracts exhibited cytotoxic effects to HDPCs.

### 3.6. LWE, SMWE, MLWE, EWE and CRWE Enhanced the ALP Level in the HDPCs

**Figure 6:**
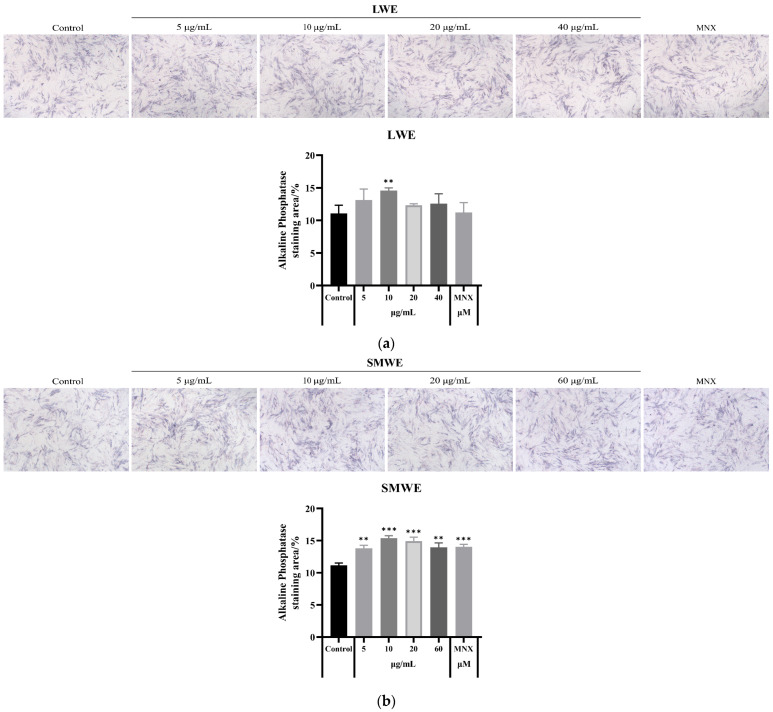

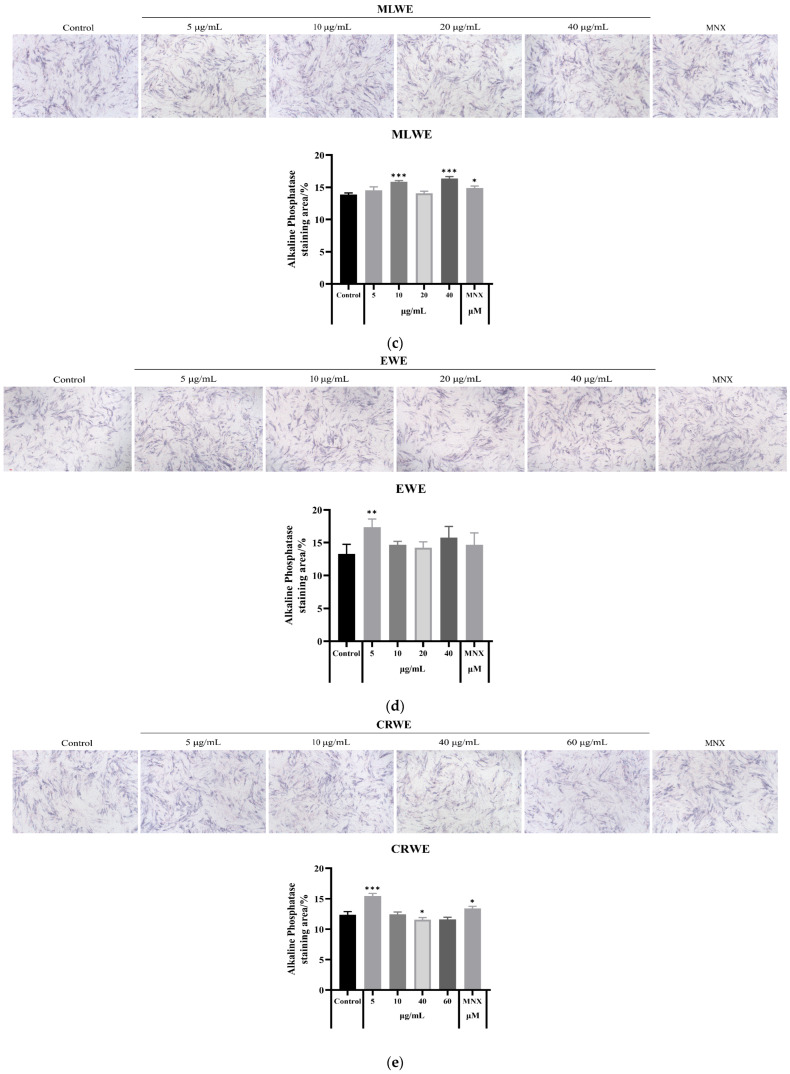
ALP expression level of HDPCs after treatment with (**a**) LWE (0, 5, 10, 20 or 40 μg/mL), (**b**) SMWE (0, 5, 10, 20 or 40 μg/mL), (**c**) MLWE (0, 5, 10, 20 or 40 μg/mL), (**d**) EWE (0, 5, 10, 20 or 40 μg/mL) and (**e**) CRWE (0, 5, 10, 40 or 60 μg/mL) (n = 4). Results are presented as mean ± standard deviation of the mean. * *p* < 0.05, ** *p* < 0.01 and *** *p* < 0.001 vs. control group.

ALP is a highly expressed dimeric protein that plays an active role in the capabilities of hair papillae and the inductive properties of hair follicles. It has been identified as one of the key markers that promote hair growth [23,24]. The expression of the ALP level in HDPCs was assessed using ALP staining with different water extracts, as shown in Figure 6. The experimental results indicate that the different water extracts significantly promote the expression of ALP compared to the control.

### 3.7. Promoting Effect of LWE, SMWE, MLWE, EWE and CRWE on Secretion of VEGF in HDPCs

**Figure 7:**
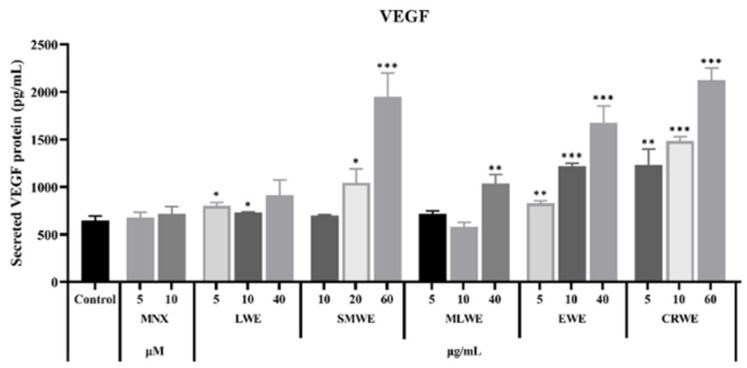
VEGF expression level of HDPCs after treatment with LWE (5, 10 or 40 μg/mL), SMWE (10, 20 or 40 μg/mL), MLWE (5, 10 or 40 μg/mL), EWE (5, 10 or 40 μg/mL), CRWE (5, 10 or 60 μg/mL) and MNX (5 or 10 μM). Results are presented as mean ± standard deviation of the mean. * *p* < 0.05, ** *p* < 0.01 and *** *p* < 0.001 vs. control group.

The formation of new blood vessels around hair follicles provides the essential nutrients and oxygen necessary for their development and growth, thereby promoting hair regeneration. VEGF is a key factor that enhances microvascular permeability and angiogenesis [24]. To investigate the effects of different water extracts on the expression of VEGF in HDPCs, we measured the secretion of VEGF in HDPCs treated with different water extracts using the ELISA assay. The results showed that after 48 h of treatment with different water extracts, the expression of VEGF in HDPCs increased to varying degrees compared to the control (Figure 7). Among them, the VEGF expression levels in the water extracts of SMWE, EWE and CRWE showed a dose-dependent increase. The high-dose experimental groups significantly (*p* < 0.001) enhanced VEGF expression, with increases of 200.95%, 159.11% and 228.3%, respectively.

### 3.8. Inhibitory Effect of LWE, SMWE, MLWE, EWE and CRWE on Expression of TGF-β1 and IL-6 in HDPCs

**Figure 8:**
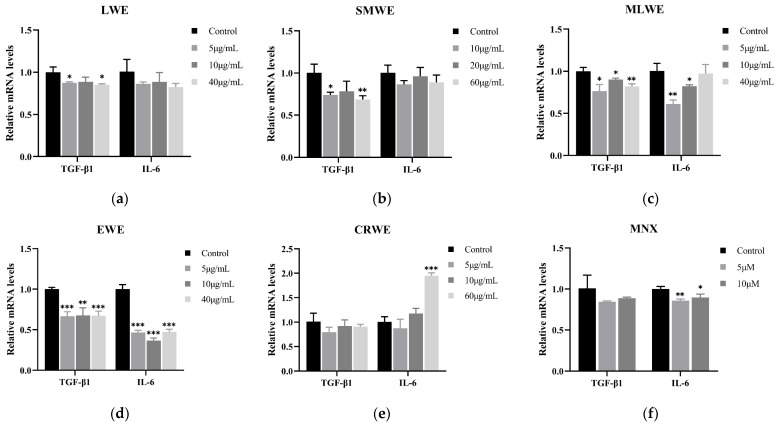
TGF-β1 and IL-6 expression level of HDPCs after treatment with (**a**) LWE (0, 5, 10 or 40 μg/mL), (**b**) SMWE (0, 10, 20 or 60 μg/mL), (**c**) MLWE (0, 5, 10 or 40 μg/mL), (**d**) EWE (0, 5, 10 or 40 μg/mL), (**e**) CRWE (0, 5, 10 or 60 μg/mL) and (**f**) MNX (0, 5 or 10 μM). Results are presented as mean ± standard deviation of the mean. * *p* < 0.05, ** *p* < 0.01 and *** *p* < 0.001 vs. control group.

The TGF-β family is a group of factors that inhibit hair growth. TGF-β1, one of its subtypes, promotes the transition of hair follicles into the regression phase by inhibiting cell proliferation and inducing apoptosis [25,26,27]. Compared to the control, all five water extracts demonstrated varying degrees of inhibition on the expression of TGF-β1 in HDPCs to varying degrees, which is shown in Figure 8. The low- and high-dose experimental groups of LWE significantly inhibited TGF-β1 expression (*p* < 0.05) compared to the control (Figure 8a). The high-dose experimental group of SMWE reduced TGF-β1 expression by 31.51% relative to the control, which was more effective than the positive control, MNX (Figure 8b,f). The low-, medium-, and high-dose experimental groups of MLWE significantly inhibited TGF-β1 expression, resulting in reductions of 23.56%, 9.91% and 18.12%, respectively (Figure 8c). The mRNA expression level of TGF-β1 in the EWE experimental group reached its lowest point at a concentration of 5 μg/mL (Figure 8d). The mRNA expression level of TGF-β1 in the CRWE experimental group decreased, but it was not statistically significant (Figure 8e). Additionally, microinflammation of hair follicles is a significant factor contributing to hair loss. IL-6 is a pro-inflammatory factor that can damage the structure of hair follicles, leading to the apoptosis of hair papilla cells [28,29]. In the experimental groups with low, medium, and high doses of LWE and SMWE, the relative expression levels of IL-6 mRNA in HDPCs were reduced, but these changes were not statistically significant (Figure 8a,b). The low, medium, and high doses of EWE significantly inhibited the expression of the inflammatory factor IL-6 (*p* < 0.001), resulting in reductions of 53.38%, 63.50% and 52.78%, respectively (Figure 8d). Additionally, treatment with MLWE and CRWE, the low-dose experimental group effectively inhibited the expression of IL-6 (Figure 8c,e). However, as the concentration of the water extracts increased, the relative expression level of IL-6 mRNA rose.

### 3.9. Promoting Effect of LWE, SMWE, MLWE, EWE and CRWE on Expression of β-Catenin in HDPCs

**Figure 9:**
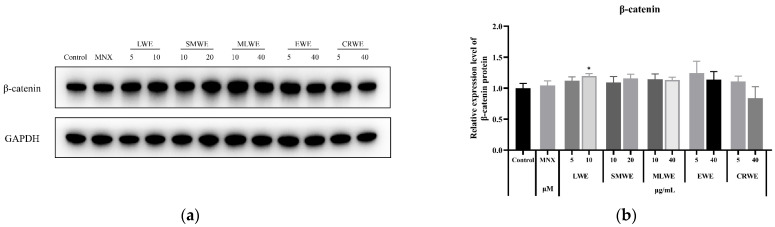
Protein expression levels of β-catenin was evaluated by western blot analysis (**a**). Results are presented as mean ± standard deviation of the mean (**b**). * *p* < 0.05 vs. control group.

The Wnt/β-catenin signaling pathway is one of the central signaling pathways involved in the transformation of the hair follicle from the telogen phase to the anagen phase. β-catenin is a key core member of this pathway and plays a crucial role in maintaining the hair follicle growth process [6]. As shown in Figure 9, the expression levels of β-catenin protein in HDPCs treated with different water extracts were determined through western blotting assay. The results indicated that, except for CRWE at 40 μg/mL, the expression levels of β-catenin were slightly elevated in response to the water extracts. Among them, the increase in β-catenin expression was dose-dependent for both LWE and SMWE.

## 4. Discussion

18-β-glycyrrhetinic acid in licorice extends the anagen phase by inhibiting the expression of TGF-β1, thereby promoting the proliferation of dermal papilla cells and outer root sheath cells [30]. According to previous reports, glycyrrhizic acid promotes the expression of β-catenin and VEGF, thereby stimulating the formation of neoplastic hair follicles on the back of mice [31]. Jin et al. [32] found that the Salvia plebeia extract activated the Wnt/β-catenin pathway by enhancing the expression of β-catenin and promoting the phosphorylation of GSK-3β. Furthermore, it increased the ratio of the anti-apoptotic proteins Bcl-2 to Bax. It activates hair follicle cells and induces an anagen phase in mice, thereby promoting hair growth. Tanshinone is the main component of Salvia plebeian. Joe et al. [33] found that tanshinone can effectively inhibit NADPH oxidase activity heightened by testosterone, thereby significantly increasing follicle length. Additionally, mulberry leaf possesses the effects of nourishing blood, moistening dryness and dispelling wind evils, which can promote hair growth [34]. Ephedra can enhance blood circulation in the scalp, improve local nutrient supply and contribute to the nourishment and health of the hair roots [35]. Malassezia is commonly present on the scalps of AGA patients [36], and tulip extract has been shown to inhibit the activity of Malassezia [37].

In this study, all water extracts showed higher expression of ALP compared to the MNX group. ALP shows high activity during hair follicle papilla development [12]. It was found that an increase in ALP activity could inhibit regression progression induced by ROS [38]. The Wnt/β-catenin signaling pathway plays a crucial role in the maintenance of the hair follicle cycle. The upregulation of β-catenin expression initiates the Wnt/β-catenin signaling cascade, promoting the proliferation and migration of hair papilla cells, resulting in the stimulation of hair growth [39].

As one of the pivotal factors influencing hair growth and longevity, studies have reported that VEGF can be produced through the activation of IGF-1 and its receptors, thereby promoting the proliferation of hair papilla cells [40,41]. In this study, LWE, SMWE, MLWE, EWE and CRWE all promoted VEGF secretion. Additionally, overexpression of VEGF can promote hair follicle growth as well as accelerate hair regrowth in mice [42].

Hair undergoes a cyclical process of growth, resting, shedding and regrowth as it progresses through the anagen, catagen and telogen phases of the hair follicle, with distinct genes exerting specific influences during each phase. TGF-β leads to the transition of hair follicles from the anagen phase to the catagen phase [32]. Previous studies have found that the inhibition of TGF-β1 expression promotes the proliferation of KC, thereby prolonging the anagen phase in mice [43]. Additionally, TGF-β2 is produced during the anagen–telogen transition and can induce apoptosis in ORC [44]. In this study, LWE, SMWE, MLWE, EWE and CRWE all inhibited TGF-β1 mRNA expression to varying degrees. The secretion of inflammatory factors can also inhibit hair growth [45]. LWE, SMWE and EWE effectively inhibited IL-6 expression. Furthermore, MLWE at concentrations of 5 μg/mL and 10 μg/mL, as well as CRWE at 5 μg/mL, were also capable of inhibiting IL-6 expression. IL-6, a paracrine factor of DPC, inhibits hair shaft elongation and accelerates the transition from anagen to telogen in mice [46].

In this study, we found that LWE, SMWE, MLWE, EWE and CRWE were able to promote hair growth to a certain extent through in vitro experiments. This finding suggests a potential source of natural plant medicines for the prevention of hair loss. However, our study has certain limitations. This research is limited to observations at the cellular level and has not been further investigated using ex vivo hair follicle models or animal models. Further research is needed to evaluate the efficacy and safety of LWE, SMWE, MLWE, EWE and CRWE in preventing hair loss.

In conclusion, our research findings suggest that LWE, SMWE, MLWE, EWE and CRWE may promote hair growth by activating the Wnt/β-catenin signaling pathway and inhibiting the TGFβ/BMP signaling pathway, thereby facilitating the transition to the anagen phase of hair growth. In addition, LWE, SMWE, MLWE and EWE can stimulate HDPCs to secrete VEGF. Furthermore, with the exception of CRWE, LWE, SMWE, MLWE and EWE may inhibit microinflammation in hair follicles by suppressing the expression of the pro-inflammatory factor IL-6. All five plant extracts demonstrated potential for combating hair loss. Therefore, LWE, SMWE, MLWE, EWE and CRWE can be added as plant extract additives in anti-hair loss products.

## Figures and Tables

**Table 1:** Primer sequences.

Primer Name	Primer Sequences
GAPDH	F: GGAGCGAGATCCCTCCAAAAT
	R: GGCTGTTGTCATACTTCTCATGG
TGF-β1	F: GCAACAATTCCTGGCGATACCTC
	R: CCTCCACGGCTCAACCACTG
IL-6	F: AGGGCTCTTCGGCAAATGTA
	R: GAAGGAATGCCCATTAACAACAA

**Table 2:** Information on the top 9 active ingredients in terms of degree value based on key pathway targets.

Number	HERB ID	Ingredient	Degree	PumChem ID	MW	Log P	H Bond Donor	H Bond Acceptor	Rotbonds
1	HBIN001991	17-beta-estradiol	13	5757	272.4	4	2	2	0
2	HBIN046831	trans-resveratrol	11	445154	228.24	3.1	3	3	2
3	HBIN028102	glycerin	9	753	92.09	−1.8	3	3	2
4	HBIN020984	citric acid	8	311	192.12	−1.7	4	7	5
5	HBIN041721	quercetin	6	5280343	302.23	1.5	5	7	1
6	HBIN040799	progesterone	5	5994	314.5	3.9	0	2	1
7	HBIN029342	hexose	4	439357	180.16	−2.6	5	6	1
8	HBIN001987	17alpha-estradiol	4	68570	272.4	4	2	2	0

**Table 3:** Information on the top 9 active ingredients in terms of degree value based on key differential lipids.

Number	HERB ID	Ingredient	Degree	PumChem ID	MW	Log P	H Bond Donor	H Bond Acceptor	Rotbonds
1	HBIN028102	glycerin	26	753	92.09	−1.8	3	3	2
2	HBIN001991	17-beta-estradiol	25	5757	272.4	4	2	2	0
3	HBIN041721	quercetin	19	5280343	302.23	1.5	5	7	1
4	HBIN029342	hexose	18	439357	180.16	−2.6	5	6	1
5	HBIN046831	trans-resveratrol	17	445154	228.24	3.1	3	3	2
6	HBIN025875	ethyl aldehyde	13	177	44.05	−0.3	0	1	0
7	HBIN001987	17alpha-estradiol	12	68570	272.4	4	2	2	0
8	HBIN020389	cholalic acid	11	221493	408.6	3.6	4	5	4

**Table 4:** The information of top 17 plant raw materials based on key pathway targets.

Number	Herb	Degree
1	Licorice	66
2	*Corydalis yanhusuo*	51
3	*Salvia miltiorrhiza*	49
4	*Scutellaria pycnoclada*	39
5	Lignum dalbergiae odoriferae	33
6	Mulberry leaf	30
7	*Artemisia annua*	30
8	Meadowrue root and rhizome	29
9	Celandine	28
10	*Peucedanum pastinacifolium*	27
11	Amur cork-tree bark	27
12	Ephedra	27
13	*Millettia pseudoracemosa*	27
14	Suberect spatholobus stem	26
15	Galangal	26
16	Rosewood heart wood	25
17	*Curcumae radix*	25

**Table 5:** The information of top 17 plant raw materials based on key differential lipids.

Number	Herb	Degree
1	Ephedra	157
2	Commiphora myrrh	146
3	Perilla frutescens	138
4	Ginger	137
5	Chrysanthemum	126
6	Salvia miltiorrhiza	124
7	Radix Bupleuri	124
8	Coriandrum sativum	120
9	Mulberry leaf	117
10	Curcumae radix	116
11	Licorice	110
12	Cinnamomi ramulus	108
13	Notopterygium incisum	107
14	Villous amomum fruit	107
15	Magnoliae flos	105
16	Manchurian wild ginger	105
17	Peppermint	102

## Data Availability

The raw data supporting the conclusions of this article will be made available by the corresponding author upon request.

## Supplementary Materials

The following supporting information can be downloaded at https://www.mdpi.com/article/10.3390/cimb47010068/s1.

## Abbreviations

The following abbreviations are used in this manuscript:

BMP	Bone morphogenetic proteins
VEGF	Vascular endothelial growth factor
TGF-β	Transforming growth factor beta
TGF-β1	Transforming growth factor beta 1
TGF-β2	Transforming growth factor beta 2
IL-6	Interleukin 6
HDPCs	Human dermal papilla cells
ORC	Outer root sheath cells
KC	Keratinocytes
