# Supplementary material for: Screening of Anti-Hair Loss Plant Raw Materials Based on Reverse Network Pharmacology and Experimental Validation
Source: Curr Issues Mol Biol. 2025 Jan 20;47(1):68. doi: 10.3390/cimb47010068 (PMC11764182; doi:10.3390/cimb47010068)
Supplement: Supplementary file 1 [file cimb-47-00068-s001.zip › cimb-3423534-supplementary.pdf]

Table S1. Information of 53 intersecting targets.

Number	Gene name	Full name
1	AR	Androgen receptor
2	CAPN1	Calpain-1 catalytic subunit
3	CDC45	Cell division control protein 45 homolog
4	CTSB	Cathepsin B
5	CYP19A1	Cytochrome P450 19A1
6	CYP26B1	Cytochrome P450 26B1
7	CYP26A1	Cytochrome P450 26A1
8	DGAT1	Diacylglycerol O-acyltransferase 1
9	EGFR	Epidermal growth factor receptor
10	DPP4	Dipeptidyl peptidase 4
11	ESR1	Estrogen receptor
12	FABP2	Fatty acid-binding protein 2
13	FGFR1	Fibroblast growth factor receptor 1
14	FKBP1A	Peptidyl-prolyl cis-trans isomerase FKBP1A
15	FPGS	Folylpolyglutamate synthase, mitochondrial
16	G6PD	Glucose-6-phosphate 1-dehydrogenase
17	GCG	Glucagon
18	HLA-A	HLA class I histocompatibility antigen
19	HMGCR	3-hydroxy-3-methylglutaryl-coenzyme A reductas
20	HSD11B1	11-beta-hydroxysteroid dehydrogenase 1
21	HSD11B2	11-beta-hydroxysteroid dehydrogenase type 2
22	HSB17B7	17-beta-hydroxysteroid dehydrogenase 7
23	ICAM1	Intercellular adhesion molecule 1
24	ITGAL	Integrin alpha-L
25	ITGB2	Integrin beta-2
26	KDM5C	Lysine-specific demethylase 5C
27	LSS	Lanosterol synthase
28	LTA4H	Leukotriene A-4 hydrolase
29	MAPK14	Mitogen-activated protein kinase 14
30	MDM2	E3 ubiquitin-protein ligase Mdm2
31	MMP2	Matrix Metallopeptidase 2
32	NR1H2	Nuclear Receptor Subfamily 1 Group H Member 2
33	PDE4A	Phosphodiesterase 4A
34	NR3C1	Nuclear Receptor Subfamily 3 Group C Member 1
35	PFKFB3	Fructose-2,6-Biphosphatase 3
36	PLG	Plasminogen
37	POLB	DNA Polymerase Beta
38	PPARA	Peroxisome Proliferator Activated Receptor Alpha
39	PPARG	Peroxisome Proliferator Activated Receptor Gamma
40	PTGDR2	Prostaglandin D2 Receptor 2
41	PTGFR	Prostaglandin F Receptor

42	PTPN1	Protein Tyrosine Phosphatase Non-Receptor Type 1
43	RARG	Retinoic Acid Receptor Gamma
44	REN	Renin
45	RXRA	Retinoid X Receptor Alpha
46	SELP	Selectin P
47	SHBG	Sex Hormone Binding Globulin
48	SLC10A2	Solute Carrier Family 10 Member 2
49	SRD5A1	Steroid 5 Alpha-Reductase 1
50	SRD5A2	Steroid 5 Alpha-Reductase 2
51	UGCG	UDP-Glucose Ceramide Glucosyltransferase
52	VDR	Vitamin D Receptor
53	ACE	Angiotensin I Converting Enzyme
